# The applicability of social cognitive career theory in predicting life satisfaction of university students: A meta-analytic path analysis

**DOI:** 10.1371/journal.pone.0237838

**Published:** 2020-08-21

**Authors:** Roziah Mohd Rasdi, Seyedali Ahrari

**Affiliations:** Faculty of Educational Studies, Universiti Putra Malaysia, Serdang, Selangor, Malaysia; Harvard University, UNITED STATES

## Abstract

Derived from the social cognitive career theory (SCCT), the present study developed a model for the empirical examination of factors affecting the life satisfaction of university students. A random-effects meta-analysis of zero-order correlations observed the results of 16 studies (20 samples, n = 7,967), and associations among the SCCT variables were examined by using a meta-analytic structural equation modeling (MASEM) according to a pooled correlation matrix. An alternative model was offered and then assessed. The findings showed a satisfactory fit of the new model as compared to the original SCCT. The results demonstrated support for the alternative model of SCCT in predicting life satisfaction. The present study suggested that researchers should embrace this alternative model when synthesizing SCCT factors. Limitations and avenues for future research were put forward for further consideration.

## Introduction

The university-to-work transition is a vital step in the creation of a job identity for graduates. During this period, some can face this transition confidently, while others experience hesitancy, insecurity, and hopelessness [1]. Pierceall and Keim [2] discovered that 87% of university students experience moderate to high levels of stress during this stage of life. This stress leads to many undesirable results such as anxiety, isolation, and low self-confidence [3], which might influence their mental health. Therefore, Lange [4] stressed that career uncertainty, as an apparent risk and fear of prospect joblessness, has a negative consequence on people’s well-being.

In this regard, previous research has emphasized that career development experience is a vital source to facilitate university students from their unclear future, tackle unfavorable working situations, and therefore increase their life satisfaction [5]. These studies also proposed that university students who are self-assured in their career orientation are satisfied with life [6]. From this standpoint, it is important to examine how students experience concomitant successes and failures at the university-to-work shift. Therefore, the present study investigates how these can affect students’ life satisfaction. For this purpose, the study draws on the concept of life satisfaction from Diener and colleagues [7], who described it as the subjective evaluation and overall cognitive judgment of life.

Life satisfaction is a main factor in the work domain, a supreme goal in human existence after basic needs, and has numerous further positive components such as being an active social player and being in good health [8]. Work and organizational psychologists are recognizing life satisfaction as an important issue as it may both cause career associated consequences and be affected by career-related aspects [9]. Results of previous research have led researchers to assume that career development is potentially linked to general views about life [10]. Therefore, life satisfaction (LS) is the desired aim for both an individual and an organization.

**Fig 1:**
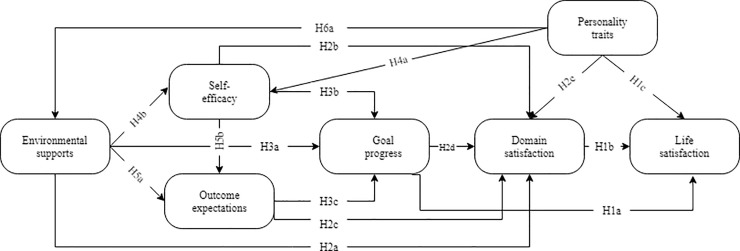
SCCT model of normative well-being of life satisfaction [20].

To date, LS of individuals, particularly university students, continues to be diminished in the career-related literature. Consequently, there is a lack of theoretical base around university students’ LS through the university-to-work process and in facilitating their career growth since some fundamental theories [11, 12] may not effectively comprise the environmental and contextual elements that contribute to the effects of career development exposure on LS [13]. As a multistage and leading career model, the social cognitive career theory (SCCT) [14] has revealed to be comprehensive in explaining the distinctive career development practices of different people [15–18]. The findings of these studies have constantly shown meaningful associations between predictor constructs in the SCCT and life satisfaction (see Fig 1). Blustein [19] affirmed SCCT as “the most important theory in career development which offers a powerful clarifying notion for scholars” (p. 350). It is proposed that human traits, social-cognitive factors, along with measurable success in other domains of life, should be expected for LS [20].

Nevertheless, the extent of the theoretical constructs of SCCT in predicting life satisfaction among university students is inadequate. Findings regarding socio-cognitive factors in predicting LS are conflicting. For instance, some have declared that personality features toward life satisfaction are more important than domain satisfaction [21], while other researchers disagreed [22]. These diverse results regarding SCCT necessitate a meta-analytic review.

A meta-analysis offers an organized tactic for studying empirical literature to synthesize results through studies. The structural equation modeling (SEM) is a technique that is normally employed for confirming whether the theory of hypothetical models fits the data [23]. The approach of merging meta-analysis and SEM could help create theory-based interferences to foster life satisfaction. Several past research has studied SCCT by using meta-analysis. Many of them have investigated a particular outcome, such as academic performance [24], choice goals [25], and career functioning [26]. Nevertheless, they examined the efficiency of SCCT variables in predicting how individuals perform actions for others’ benefits instead of individual benefits, such as life satisfaction. Thus, this study aims to test SCCT and how it can predict the life satisfaction of university students by using meta-analytic path analysis.

### Literature review

SCCT is a model proposed by Lent [14] to clarify the conditions in which: (a) career hobbies are established; (b) academic and vocational options are chosen; (c) career determination is performed. It has received extensive consideration from researchers whereby many of them have placed the model on the career growth of school and university students [27]. The key notion has been that though career choice actions are performed through the lifetime, the concepts are most noticeable through the late teenage years and early adulthood when people are planning to join the workforce [14]. For this reason, not much attention has been paid to the value of this theory to predict outcomes for youths who are in the middle of the university-to-work transition. The university-to-work transition refers to on-the-job training, vocational training, service-learning agreements, or other programs designed to prepare university students to enter the workforce.

Lent and Brown [28] later extended SCCT to a satisfaction model. While the extended SCCT employs the term work satisfaction, this is meant to generally comprise other forms of satisfaction as well. Lent, Brown, and Hackett [29] suggested that SCCT, with its attention to both individual and background variables, can be a useful model to describe the university-to-work transition. The SCCT satisfaction model also includes self-efficacy (SE), outcome expectations (OEs), goal progress (GP), personality traits (PTs), domain satisfaction (DS), and environmental supports (ESs), which predict life satisfaction. Besides, this point needs to be reminded that, self-efficacy means a personal judgment of "how well one can execute courses of action required to deal with future situations [100]. Also, domain satisfaction reflects the extent to which objective conditions in a particular area of life match individuals’ respective needs or aspirations [79].

On the other hand, Lent and Brown [30] stated that general measures of SCCT variables may have limited utility, in that these variables require to be deliberated in domain-specific contexts. Thus, they warned academics to utilize domain-specific measures in any examinations of extended SCCT.

Previous studies found that life satisfaction was significantly associated with DS in the context of university-to-work transition, thus providing logic for embracing life satisfaction as an outcome construct [31]. Lent and Brown [28] mentioned that Heller, Watson, and Ilies’ [32] research is one of the bases for the satisfaction model. According to Heller and colleagues [32], the relative pros of top-down (person-based) or bottom-up (situation-based) approaches toward explaining subjective well-being in binary routes are as follows: (a) they studied associations between PTs, DS measures, and life satisfaction; and (b) they created and examined three rival hypothetical models. The initial model was named as “straight effects” top-down model, wherein PTs were each linked directly to DS and life satisfaction. The second model, named the personality top-down model, illustrated the PTs as having individual links directly to life satisfaction. Lastly, the third model, the combining model, offered direct associations between PTs with every satisfaction domain (marital, job, and life). Besides, DS linked directly to life satisfaction. This is the model as a blend of the top-down and bottom-up approaches toward describing subjective well-being [32]. Their study supported the notion that life satisfaction and DS are related constructs.

### Goal progress, domain satisfaction, personality traits, and life satisfaction

Discussions on GP and DS on life satisfaction of university students have been directly or indirectly debated since the original work of Lent and Brown [30]. GP represents a third type of cognitive variable. According to Latham, Mawritz, and Locke [33], having goals and experiencing growth toward one’s goals directs to bigger stages of satisfaction. Sheldon and Kasser’s [34] study supported the relationship between perceived GP and satisfaction. For university students, academic GP was predictive of enrolment and persistence in academic majors [35]. Lent [20] stated that goals may encourage an individual’s feeling of satisfaction by activating optimistic responses in reaction to his/her perceived progress on an appreciated goal. On the contrary, the lack of such support, or the existence of background barriers, is possible to block GP and decrease life satisfaction [30]. Besides, Lent [36] showed that DS (e.g., academic satisfaction) was the most consistent predictor of life satisfaction. Previous literature has commonly believed that an additive association among DS and life satisfaction does exist [37]. Lent and Brown [28] characterized life satisfaction as having “trait-like features” (p. 243). Thus, they conceptualized PTs as inputs to the satisfaction model. PTs can influence people’s life satisfaction [38]. Therefore:

**Hypothesis 1a**: GP is positively linked to life satisfaction.
**Hypothesis 1b:** DS is positively linked to life satisfaction.
**Hypothesis 1c:** PTs are positively linked to life satisfaction.

### Environmental supports, self-efficacy, outcome expectations, goal progress, personality traits, and domain satisfaction

ESs as contextual affordances can facilitate an individual’s vocational option and growth [35]. SCCT is concerned with ESs as a predictor of DS. Gibbons and Shoffner [39] acclaimed SCCT as most promising as it embraces constructs that explain disparities in environmental opportunities along with individuals’ beliefs about the environment. Researchers have found support for including ESs in the social cognitive model of the domain in university students’ sample [40]. According to Lent and Brown [30], ESs as reinforcement, modeling, and positive feedback provide assets to individuals that can increase the sense of satisfaction [20]. Besides, persons differ in their SE regarding the behaviors needed in several academic and career domains. SE beliefs are pretty dynamic and are specific to specific activity domains. SE as a fundamental part of one’s life provides a vital advantage in terms of social and career development [41]. Lent’s [20] research supported the relationship between OEs and DS. The SCCT model forecasts satisfaction in the social domain through straight routes from the combined effects of social cognitive constructs [36]. Further study is required to define the association between OEs and DS. Lent [36] discovered that GP was predictive of DS. According to Wiese and Freund [42], career development depends partly on external factors but is also determined by personal goals. The meta-analysis of Klug and Maier [43] revealed a strong connection between GP and satisfaction in a particular domain. Moreover, Lent [44] proposed that factors like PTs influence DS. Lent’s normative model of well-being (see Fig 1) offers cognitive, behavioral, environmental, and personality elements that regulate individuals’ DS [36, 45]. Hence:

**Hypothesis 2a:** ESs are positively linked to DS.
**Hypothesis 2b:** SE is positively linked to DS.
**Hypothesis 2c:** OEs are positively linked to DS.
**Hypothesis 2d:** GP is positively linked to DS.
**Hypothesis 2e:** PTs are positively linked to DS.

### Environmental supports, self-efficacy, outcome expectations, and goal progress

Lent [46] uncovered that ESs, which comprise goal-relevant assets, modeling, and inspiration, is associated with affecting GP (*r* = .23). Furthermore, Lent [14] found that SE will directly affect the kinds of interests that a person will grow. Later, Lent [47] stated that SE is a predictor of GP for undergraduates because they anticipate to obtain favorable results when pursuing actions at which they think are effective. Higher SE appraisals are also associated with GP, regardless of barriers. Moreover, OEs also play a key role in motivating individuals toward their goals. According to the SCCT model, OEs and SE together shape motivation [14]. Satisfaction can be viewed as somewhat a function of individuals’ positive OEs about that possible result from pursuing an esteemed goal [48]. Therefore:

**Hypothesis 3a:** ESs are positively linked to GP.
**Hypothesis 3b:** SE is positively linked to GP.
**Hypothesis 3c:** OEs are positively linked to GP.

### Personality traits, environmental supports, and self-efficacy

In the career development context, SE refers to persons’ confidence in their skill to succeed in creating academic or vocational choices. Scholars highlighted the vitality of examining the initial phase of career development because it is an important stage when PTs and career SE are founded [49]. Thus, they have started to call for the combination of PTs into any model assessing the backgrounds of academic and career options. PTs may influence vocational confidence in a parallel way to interests [14]. Although limited studies have examined the relationships between PTs and the vocational and career constructs [50], it is still difficult to illustrate strong deductions regarding the effects of PTs due to the use of varying scales. More recently, scholars have pursued to study if and how PTs influence the cognitive constructs involved in the career development progressions of SE [22]. In this context, PTs are also considered as direct antecedents of SE in which both PTs and SE have proven to be significant antecedents of academic and career success [51]. Studies showed that ESs are indirectly linked to life satisfaction through SE and OEs [28]. In their longitudinal study, Hou, Wu, and Liu [52] found that environmental and social supports predict SE among Chinese students. Therefore:

**Hypothesis 4a:** PTs are positively linked to SE.
**Hypothesis 4b:** ESs are positively linked to SE.

### Environmental supports, self-efficacy, and outcome expectations

OEs are individuals’ attitudes about the effects of their behaviors and can be influenced by previous experiences, skills, and performance in addition to social supports [14]. According to Franco [53], OEs do not happen in a vacuum and socio-cultural environments may affect these expectations. In the SCCT model, OEs are informed by vicarious environmental factors (e.g., social support) and they later play a central role in predicting career options [54]. Kelly [27] found that ESs are unique contributors to OEs. Hui, Yuen, and Chen’s study [55] also validated the link between ESs and OEs. According to Garriott [56], ES predicted college OEs in first-and non-first-generation students. Bandura [57] posited that SE is another significant source of OEs, which means higher confidence in a person’s skill of completing certain tasks would result in more positive perceptions of the outcomes. According to Ali, McWhirter, and Chronister [58], vocational/educational SE beliefs are accounted for a meaningful total of variance in vocational and educational OEs. Therefore:

**Hypothesis 5a:** ESs are positively related to OEs.
**Hypothesis 5b:** SE is positively related to OEs.

### Personality traits and environmental supports

PTs are unique qualities that are the epitome of an individual. They are individuals’ habitual patterns of behavior, temperament, and emotion [59]. According to the social cognitive theory and symbolic interactionism, humans are not passive agents; however, as these traditions also suggest, individuals’ PTs are actively shaped by the environment [60]. Previous studies similarly addressed the roles of PTs and ESs, such as background experiences [61]. PTs in the SCCT model may comprise characteristics like trait positive/negative effects and are hypothesized to predict ESs [56]. Furthermore, PTs have been shown to predict ESs [44]. Longitudinal investigations have provided support for this relationship [62]. It means that PTs can provide the motivational impulses or the motivational blocks to use or not to use environmental and social supports and thus to improve or reduce performance [63]. Therefore:

**Hypothesis 6a:** PTs are positively related to ESs.

## Materials and methods

### Search strategy for identifying studies

The authors of the present study perused PsycNet, ProQuest, and ScienceDirect, to find related research published between January 1, 2004, and December 31, 2018. The combination of words employed to search these records were life, academic, and domain satisfaction, outcome expectations, self-efficacy, environmental and social supports, and social cognitive career theory. Studies met the inclusion criteria if they: (a) employed the SCCT as a theoretical basis, and (b) supplied quantitative data allowing for the calculation of correlation (*r*) and/or effect sizes among the SCCT variables.

**Fig 2:**
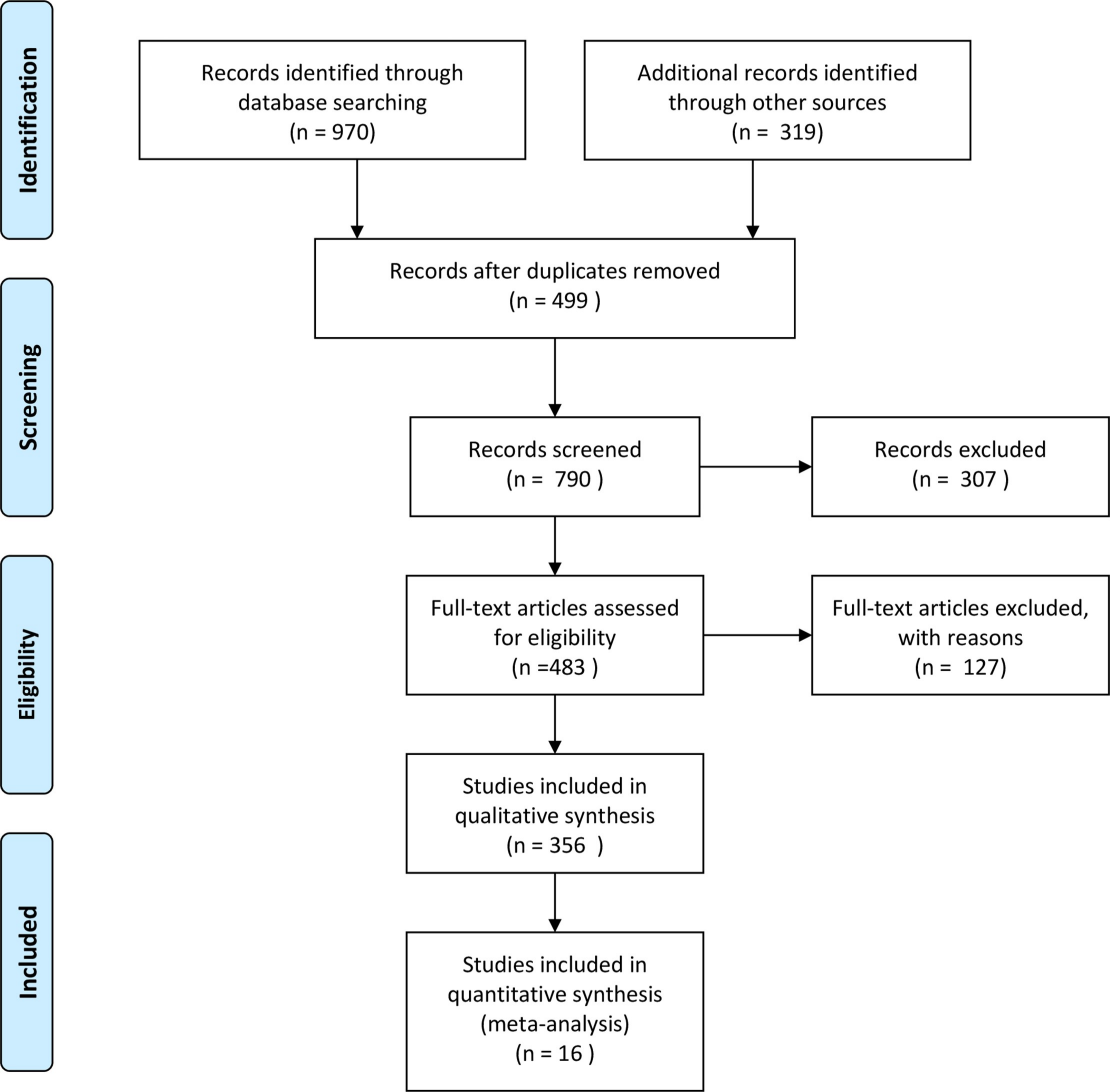
Flow chart of the study selection process in this meta-analysis.

As well as exploring journal databases, additional search tactics were used to enlarge the number of studies. The authors investigated the websites of well-known journals with a history of releasing higher education and career studies. They also inspected article references and searched for authors by name. Abstracts were assessed according to the inclusion criteria. First, they excluded duplicated research, studies that involved school students (i.e., young people before entering higher education) as participants, and qualitative studies. Second, studies were excluded if they did not meet all of the inclusion standards, or if inadequate data were available for calculation. Sixteen studies (20 samples) were chosen for the meta-analytic path analysis (see Fig 2). Finally, Cooper’s [64] method of the unstructured search was employed by using Google Scholar and Google for identifying more relevant studies (see Table 1).

**Table 1:** Strategy for literature search.

Database	Search terms	Number of studies
PsycNet	SCCT*, university students*, and life satisfaction	189
ProQuest	SCCT*, university students*, and life satisfaction	456
ScienceDirect	SCCT*, university students*, and life satisfaction	325
Other databases (Google Scholar, Google, etc.)	SCCT*, university students*, and life satisfaction	319
Duplicates removed		499
Record screened		790
Records excluded		307
Full-text studies assessed for eligibility		483
Full-text articles excluded, with reasons		127
Studies included in the qualitative synthesis		356
ULTIMATE study collection (once assessing the quality)		16

* Indicates that any word starting with this term would be used in the study.

### Data extraction

Data were obtained by a predesigned protocol that was based on the ‘Joanna Briggs Institute’ [65] and ‘Preferred Reporting Items for Systematic Reviews and Meta-analysis’ (PRISMA) regulations [66]. It was re-examined and filtered by an expert group including a higher education expert and a career development university lecturer. The obtained outcome data comprised a correlation matrix of SCCT variables, which were ESs, SE, OEs, GP, DS, PTs, and life satisfaction. The sample size (*n*) of every study and the observed association of SCCT variables were gathered to execute the meta-analytic path analysis.

### Quality assessment

The sixteen studies that achieved the inclusion criteria were evaluated individually by two researchers for methodology characteristic by the ‘Quality Assessment and Validity Tool for Correlational Studies’ (QAVTCS) [67]. The quality assessment tool was employed to check four key parts of the study: design, sample selection, instrument, and data analysis. Thirteen standards were assessed, with a total of fourteen possible scores. Build on the designated score, studies were categorized as low (0–4), moderate (5–9), or high (10–14) quality. Inconsistencies in the scores were settled after the debate between the two researchers. This resulted in sixteen studies rated as high. The rest did not achieve most of the criteria listed in the QAVTCS, so it was scored as 3 which was of low-level quality and was omitted from additional review. Therefore, all sixteen studies were kept for analysis (see Table 2).

**Table 2:** Summary of quality assessment.

No.	**Criteria**	Lent [68]	Silva [69]	Işık [70]	Lee [71]	Zalazar-Jaime [72]	Truong & Miller [73]	Antl [74]	Ezeofor [75]
**1**	Future studies	1	1	1	1	1	1	1	1
**2**	Probably sampling	1	1	1	1	1	1	1	1
**3**	Suitable size of the sample	0	1	1	1	1	0	1	0
**4**	Sample obtained from more than one location	0	0	0	1	1	1	1	0
**5**	Privacy preserved	1	1	1	1	1	1	1	1
**6**	Response rate >60%	1	1	1	1	1	1	1	1
**7**	Reliable measurement of result (s)	1	1	1	1	1	1	1	1
**8**	Valid measure of result (s)	1	1	1	1	1	1	1	1
**9**	Valid measure of independent variables	1	1	1	1	1	1	1	1
**10**	Life satisfaction internal consistency	2	2	2	2	2	2	2	2
**11**	Theory driven	1	1	1	1	1	1	1	1
**12**	Correlation analysis for multiple effects	1	1	1	1	1	1	1	1
**13**	Outliers management	0	0	1	1	0	0	1	1
	Total	11	12	13	14	13	12	14	12
No.	**Criteria**	Sheu [76]	Sheu [77]	Jezzi, [78]	Ojeda [40]	Lent [79]	Lent [80]	Bergin & Jimmieson [81]	Garriott [56]
**1**	Future studies	1	1	1	1	1	1	1	1
**2**	Probably sampling	1	1	1	1	1	1	1	1
**3**	Suitable size of the sample	1	1	1	1	1	1	0	1
**4**	Sample obtained from more than one location	1	1	1	1	0	1	1	0
**5**	Privacy preserved	1	1	1	1	1	1	1	1
**6**	Response rate >60%	1	1	1	1	1	1	1	1
**7**	Reliable measurement of result (s)	1	1	1	1	1	1	1	1
**8**	Valid measure of result (s)	1	1	1	1	1	1	1	1
**9**	Valid measure of independent variables	1	1	1	1	1	1	1	1
**10**	Life satisfaction internal consistency	2	2	2	2	2	2	2	2
**11**	Theory driven	1	1	1	1	1	1	1	1
**12**	Correlation analysis for multiple effects	1	1	1	1	1	1	1	1
**13**	Outliers management	1	0	1	1	1	0	1	1
	Total	14	13	14	14	13	13	13	13

### Data abstraction

The present study utilized a strategy for data abstraction. The subsequent data were obtained: authors, publication year, aim, country, variables, sample size, and mean age (see Table 3 for further detail).

**Table 3:** A short review of included studies for the meta-analysis processes.

No.	Author(*s*)	Year	Type	Aim	Country	Sample	Variable(s) (M /*SD*)	Sample size	Mean age
**1**	Lent [68]	2005a	JA	To test the predictions specific by the integrative social cognitive model	USA	University students at a large eastern U.S. university	SE, OEs, GP, ESs, DS, LS (6.74/1.81, 7.05/1.11, 3.54/.66, 3.66/.65, 4.04/.86, 5/1.29)	177	18.93
**2**	Lent [68]	2005b	JA	–	USA	–	SE, GP, ESs, DS, PTs, LS (4.22/.80, 3.47/1.02, 3.71/.71, 4.72/1.37, 4.69/1.32, 4.69/1.32)	299	–
**3**	Silva [69]	2010a	CP	To understand the procedure of academic satisfaction in European and Mozambican Portuguese speaking learners	Portugal	Portuguese College Students	SE, GP, ESs, PTs, LS (34.9/1.87, 28.4/3.51, 36.7/2.65, 40.4/3.01, 25.4/2.26)	305	26.49
**4**	Silva [69]	2010b	CP	–	Portugal	Mozambican college Students	SE, GP, ESs, PTs, LS (31.2/5.69, 33/4.05, 36.8/4.75, 45.9/4.91, 22.6/5.81)	465	–
**5**	Işık [70]	2018	JA	To test social cognitive model of well-being in Turkish students	Turkey	College students	SE, GP, ESs, OEs, DS, PTs, LS (6.86/1.36, 3.87/.71, 3.87/.76, 7.47/1.46, 4.04/.81, 3.26/.76, 5.26/1.14)	303	19.67
**6**	Lee [71]	2016	JA	To mature a complex Negative OEs Scale in the engineering domain	USA	University students in a large public university in the Southwest	SE, ESs, DS, OEs	1,187	21.26
**7**	Zalazar-Jaime [72]	2017	JA	To test the academic satisfaction model in a first-year university student	Argentina	First-year university students	SE, ESs, GP, DS, PTs (33.52/13.41, 34.04/5.31, 49.25/11.08, 57.33/8.49, 34.26/6.59)	682	20.91
**8**	Truong & Miller [73]	2017	JA	To examine how contextual factors linked to the academic experiences university students	USA	Southeast Asian American college students	SE, ESs, OEs, GP, DS (7.83/1.69, 3.78/1.09, 7.38/1.33, 3.67/.69, 3.95/.62)	111	21.44
**9**	Antl [74]	2011	TE	To assess the moderating role of SE in the relationship between self-concordance and GP and subjective well-being	Canada	Undergraduates	SE, PTs, LS, GP (5.11/1.08, 4.56/1.42, 4.15/1.49, 4.86/1.70)	189	19.59
**10**	Ezeofor [75]	2013	TE	To test how self-construal is related to the social cognitive predictors of academic satisfaction	USA	Undergraduates	SE, ES, OE, GP, DS (7.52/1.13, 3.86/.74, 7.22/1.45, 3.78/.84, 3.96/.77)	174	21.1
**11**	Sheu [76]	2014a	JA	To test the cross-cultural validity of a modified version of SCCT normative well-being model	Taiwan	University students	SE, ES, OE, GP, DS, LS (5.89/1.12, 3.54/.53, 5.94/1.18, 3.21/.71, 3.67/.65, 4.43/1.21)	317	20.53
**12**	Sheu [76]	2014b	JA	-	Singapore	University students	SE, ES, OE, GP, DS, LS (5.89/1.12, 3.54/.53, 5.94/1.18, 3.21/.71, 3.67/.65, 4.43/1.21)	259	21.12
**13**	Sheu et al. [77]	2017	JA	To test a changed academic satisfaction model based on SCCT	China	University students	SE, ES, OE, GP, DS, LS (6.43/1.28, 3.37/.57, 6.09/1.66, 3.22/.64, 3.38/.97, 4.13/1.35)	757	20.92
**14**	Jezzi [78]	2016	TE	To examine a modified social cognitive model of domain satisfaction	USA	University students	SE, ES, OE, GP, DS (6.05/1.48, 4.24/.64, 6.88/1.44, 3.71/.81, 3.94/.74)	454	19.92
**15**	Ojeda [40]	2009	TE	To test the academic and life satisfaction	USA	University Students	SE, OE, GP, DS, PT, LS (6.23/1, 8.92/1, 3.89/.64, 4.21/.57, 4.30/.55, 5.18/1.27)	460	21.53
**16**	Lent [79]	2017a	JA	To expand research on the SCCT of well-being model by testing its validity in a new setting	Spain	University students	SE, ES, GP, DS, PT, LS (7.28/.81, 4.01/.41, 3.76/.47, 3.75/.57, 3.75/.44, 5.17/1.02)	373	
**17**	Lent [80]	2018	JA	To assess a form of the well-being model in a South American sample or to compare its value relative to a European sample	Brazil and Portugal	University students	SE, ES, GP, DS, PT, LS (6.69/1.28, 3.78/.49, 3.57/.60, 3.79/.56, 3.48/.59, 4.95/1.05)	706	
**18**	Bergin & Jimmieson [81]	2017a	JA	To examine the link between university OEs and student adjustment	Australia	Business students	SE, OE, DS (5.48/.95, 5.59/1.06, 2.61/.76)	135	22.85
**19**	Bergin & Jimmieson [81]	2017b	JA	-	-	-	SE, OE, DS (5.59/.77, 5.68/1, 2.71/.71)	200	
**20**	Garriott [56]	2015	JA	To assess social cognitive model of normative well-being	USA	College students	SE, ES, OE, GP, DS, PT, LS (8.01/1.22, 4.24/.52, 7.86/1.24, 4.15/.60, 4.24/.58, 3.82/.60, 5.10/1.24)	414	-

JA = Journal article, TE = Thesis, CP = Conference paper. ESs = Environmental supports; PTs = Personality traits; SE = Self-efficacy; OEs = Outcome expectations; GP = Goal progress; DS = Domain satisfaction; and LS = Life satisfaction.

## Data analysis

### Meta-analytic method

In a meta-analysis, a summary effect is provided that explains the overall trend. An important subject is then the selection among fixed or random-effects models. As found by Field and Gillett [82], scholars must decide on the proper model earlier based on the included studies and the preferred deductions. According to Borenstein [83], the random-effects model permits that the true effect size may differ from study to study (normally distributed). Particularly, random-effect models are more suitable when studies are carried out by diverse academics in different contexts so that effect sizes can vary randomly [84–86]. By contrast, the fixed-effect model depends on the notion that all researches in the meta-analysis have a shared effect size. In this study, a random-effects model was then used since most of the included studies were executed separately, with several samples drawn from various respondents (see Table 4). Furthermore, the researchers managed to conduct heterogeneity tests of effect sizes through their studies, such as 95% confidence interval (CI), Cochran's *Q* statistic, and *I*^*2*^ statistic [87]. The 95% CI of every estimate was made around the true score correlation. In particular, this study used the *Q* statistic and the I2 statistic, as *I*^*2*^ was more appropriate for meta-analyses with fewer studies [88]. The *I*^*2*^ statistic was employed to determine the degree of heterogeneity. The estimated heterogeneity variances explained in Table 5. The range of *I*^*2*^ is from 79.297 to 94.891. This proposes that the correlations are fairly heterogeneous.

**Table 4:** Raw correlation coefficient and two-tailed p-value included for the meta-analytic processes.

No.	Study	ES-PT	ES-SE	ES-OE	ES-GP	ES-DS	SE-OE	SE-PT	SE-GP	SE-DS	OE-GP	OE-DS	GP-DS	GP-LS	PT-DS	PT-LS	DS-LS
**1**	Lent a	.41	.30	.40	.26	.45	.39	.49	.61	.56	.32	.41	.61	.30	.46	.47	.40
**2**	Lent b	.13	.13	-	.03	.11	-	.18	.46	.46	-	-	.74	.29	.14	.05	.40
**3**	Silva a	.43**	.09	-	-.06	-	-	.05	.09	-	-	-	-	-.02	-	.65**	-
**4**	Silva b	.34**	.23**	-	.35**	-	-	.21**	.29**	-	-	-	-	.26**	-	.43**	-
**5**	Işık	.35	.46	.61	.58	.57	.62	.34	.62	.56	.60	.61	.63	.48	.35	.40	.51
**6**	Lee	-	.32*	.45	-	.46*	.37*	-	-	.50*	-	.48*	-	-	-	-	-
**7**	Zalazar-Jaime	.21**	-	-	.27**	.46**	-	-	-	-	-	-	.37**	-	.38**	-	-
**8**	Truong & Miller	-	.14	.15	.19**	-	-	-	.64**	.22	-.12	.24**	.36**	-	-	-	-
**9**	Antl	-	-	-	-	-	-	.40**	.31**	-	-	-	-	.33**	-	.76**	-
**10**	Ezeofor	-	.37**	.39**	.34**	.38**	.36**	-	.70**	.62**	.37**	.69**	.51**	-	-	-	-
**11**	Sheu a	-	.49	.49	-	.53	.28	-	.80	-	.11	-	.39	-	-	-	.42
**12**	Sheu b	-	.47	.24	.18	.71	.46	-	.69	-	-	-	-	-	-	-	.23
**13**	Sheu	-	.39	.40	-	.58	.16	-	.73	-	-	-	.19	-	-	-	.51
**14**	Jezzi	.34	.64	.52	.68	.58	.64	.46	.68	.58	.48	.42	.67	-	.49	-	-
**15**	Ojeda	-	-	-	-	-	.41**	.54**	.47**	.45**	.16**	.30**	.54**	.18**	.39**	.26**	.32**
**16**	Lent	.24	.22	-	.23	.41	-	.50	.51	.39	-	-	.40	.23	.44	.34	.39
**17**	Lent	.41	.40	-	.37	.54	-	.51	.57	.51	-	-	.51	.41	.57	.52	.51
**18**	Bergin & Jimmieson a	-	-	-	-	-	.86	-	-	.74	-	.05	-	-	-	-	-
**19**	Bergin & Jimmieson b	-	-	-	-	-	.87	-	-	.81	-	.12	-	-	-	-	-
**20**	Garriott	.43	.48	.51	.36	.53	.46	.52	.55	.47	.35	.50	.51	.33	.43	.43	.36

Correlation coefficient (r) and the two-tailed *p*-value that was not reported by the studies are marked as a dash (–). Significant levels considered from the original analyses (significant levels are not stated if the studies did not report them). OEs = outcome expectations; GP = Goal progress; ESs = Environmental supports; SE = self-efficacy; DS = Domain satisfaction; and LS = Life satisfaction.

* *p* < .05.

** *p* < .01.

*** *p* < .001.

**Table 5:** Random effects average correlation and heterogeneity statistics for SCCT constructs and life satisfaction.

*Associations*	*K*	*N*	*r*^*+*^	*CI 95% LI*	*Q*	*I*^*2*^ *(LI-UI)*
ES-PT	10	4178	.331	.267-.392	45.871	80.380
ES-SE	15	6301	.*336*	.276-.428	163.471	91.436
ES-OE	10	4153	.*448*	.367-.495	53.298	83.114
ES-GP	13	4722	.*331*	.186-.419	234.896	94.891
ES-DS	13	6102	.*502*	.433-.567	122.200	90.180
SE-OE	12	4377	.*451*	.397-.644	385.254	97.145
SE-PT	11	4145	.*408*	.*294-*.*482*	*129*.*533*	*92*.*280*
SE-GP	16	5763	.573	.475-.647	348.816	95.700
SE-DS	13	4993	.522	.477-.605	118.744	89.894
OE-GP	8	2410	.*328*	.150-.440	107.228	93.472
OE-DS	10	3615	.*437*	.298-.502	112.461	91.997
GP-DS	13	5227	.*482*	.415-.591	221.336	94.578
GP-LS	10	3691	.*291*	.198-.365	68.379	86.838
PT-DS	9	3868	.428	.335-.485	63.928	87.486
PT-LS	10	3691	.436	.331-.555	164.753	94.537
DS-LS	10	4065	.428	.352-.468	43.472	79.297

N = total sample size; K = number of effect sizes included in the meta-analysis procedures; CI = confidence interval; Q and I^2^ = tests of heterogeneity; r^+^ = random effects average correlation; ES = Environmental support; PT = Personality traits; SE = Self-efficacy; OE = Outcome expectations; GP = Goal progress; DS = Domain satisfaction; and LS = Life satisfaction. *p < 0.05. **p < 0.01. ***p < 0.001.

^a^ A true effect may not happen as the corresponding 95% CI includes 0.

^b^ Effect size was attained from one study only; no CI could be created.

### Path analysis

In running a path analysis by maximum-likelihood estimate, the authors used the created correlation matrix. Consequently, means and standard deviations for each construct were set to 0 and 1, respectively. According to the revealed corrected meta-analyzed correlations, the meta-analytic path analyses by AMOS 23.0 software were conducted to assess SCCT and the antecedents of life satisfaction. As in past meta-analyses that have also employed additional path analyses [89], the harmonic mean of the sample sizes underpinning each effect size described in the path models was used as the input sample size. Model fit can be evaluated by goodness-of-fit indices; like chi-square/degree of freedom ratio (CMIN/DF), comparative fit index (CFI), goodness-of-fit index (GFI), normed fit index (NFI), and root mean square error of approximation (RMSEA).

## Results

### Study characteristics

Built on this study’s criteria, sixteen studies (20 samples) in the datasets that met the inclusion criteria provided a total of 7,967 respondents. Altogether, four dissertations, one conference paper, and eleven journal articles met the study’s criteria. The oldest study included in this research was published in 2005, while the latest study was printed in 2018. The included research were from eleven countries, where the majority of them were conducted in the United States (US) (*k* = 8). The sample sizes ranged from 111 [73] and 1,187 [71].

### Total relationship between SCCT constructs

The table below shows the correlation between the six constructs of SCCT. Every average weighted correlation (*r*^+^) was meaningfully varied from zero (*p* < .001). Through the studies, life satisfaction had a low correlation with GP (*r*^+^ = .291). All 16 correlations revealed significant heterogeneity between studies, with all *Q* statistics being significant. The relationship strength between SCCT constructs was low to medium, with an effect size ranging from .150 to .647. All dimensions of SCCT, though, were significantly linked (i.e., 95% CI excluded 0). The *I*^2^ statistics proposed that the correlations between all SCCT constructs proposed high heterogeneity, as stated by Higgins [90].

### Meta-analysis correlation matrix

The results were organized into a correlation matrix (see Table 6), which facilitated to create a base for subsequent path analyses. The associations between life satisfaction and three SCCT constructs were positive (ranging from 0.291 to 0.436), while the correlations of all SCCT constructs were positive. Thus, life satisfaction revealed a low to medium association with three SCCT constructs, i.e., DS, PT, and GP.

**Table 6:** Meta-analysis correlation matrix among SCCT constructs.

	1	2	3	4	5	6	7
**1. LS**		10/4065	10/3691	10/3691	–	–	–
**2. DS**	.428 10 4065	–					
**3. PTs**	.436 10 3691	.428 9 3868	–				
**4. GP**	.291 10 3691	.482 13 5227	–	–			
**5. OEs**	–	.437 10 3615	–	.328 8 2410	–		
**6. SE**	–	.522 13 4993	.408 11 4145	.578 16 5763	.451 12 4377	–	
**7. ESs**	–	.502 13 6102	.331 10 4178	.331 13 4722	.448 10 4153	.336 15 6301	–

The diagonal of weighted correlations are shown in the cells above. The diagonal of the number of studies (*K*) and the pooled sample sizes have displayed the cells below.

ESs = Environmental supports; PTs = Personality traits; SE = Self-efficacy; OEs = Outcome expectations; GP = Goal progress; DS = Domain satisfaction; and LS = Life satisfaction. *p < 0.05. **p < 0.01. ***p < 0.001.

### Path analysis

The study examined the SCCT model by using MASEM. Confidence intervals from the meta-analytic correlation matrix are exhibited in Table 5. For model fit, Kline [91] inspired the use of model fit indices, with chi-square/degree of freedom ratio (CMIN/DF), comparative-fit index (CFI), goodness-of-fit index (GFI), and normed fit index (NFI). A rule of thumb for the fit indices is that values at 0.90 or above show acceptable fit [92]. Moreover, the model may be considered as satisfactory if the root mean square error of approximation (RMSEA) is between 0.03 and 0.08.

### Structural model

**Fig 3:**
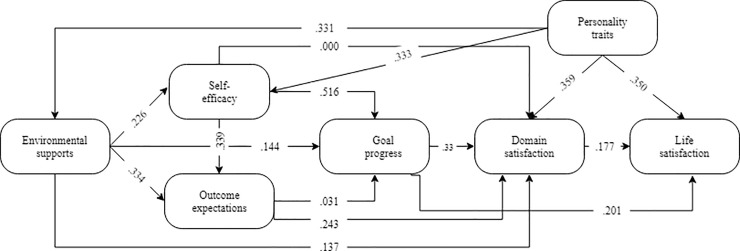
Path analysis for Model I. All paths are significant at *p* < .01.

The fit indices of the original SCCT structural model (Model I) were not satisfactory and did not fit the data across most fit indices, CMIN/DF = 4718.698, p < 0.01, IFI = .668, CFI = .666, NFI = .654, GFI = .841, and RMSEA = .473. As specified by Kline [92], the model suggested an unsuitable fit for the model. After removing GP and OEs, the second model (Model II) includes ESs, SE, DS, PTs, and life satisfaction. As revealed in Table 7, the model was satisfactory and considered as acceptable. This model offered better fit indices: CMIN/DF = 3102.582, *p* < 0.01, IFI = 0.903, CFI = 0.903, NFI = .904, GFI = .907, and RMSEA = 0.078. Following Kline [92], the model suggested an acceptable and appropriate fit (see Fig 3).

**Table 7:** Fit indices of SEM models.

Model	CMIN/DF	IFI	CFI	NFI	GFI	RMSEA
Model I	4718.698	.668	.666	.654	.841	.473
Model II	3102.582	.903	.903	.904	.907	.078

**Fig 4:**
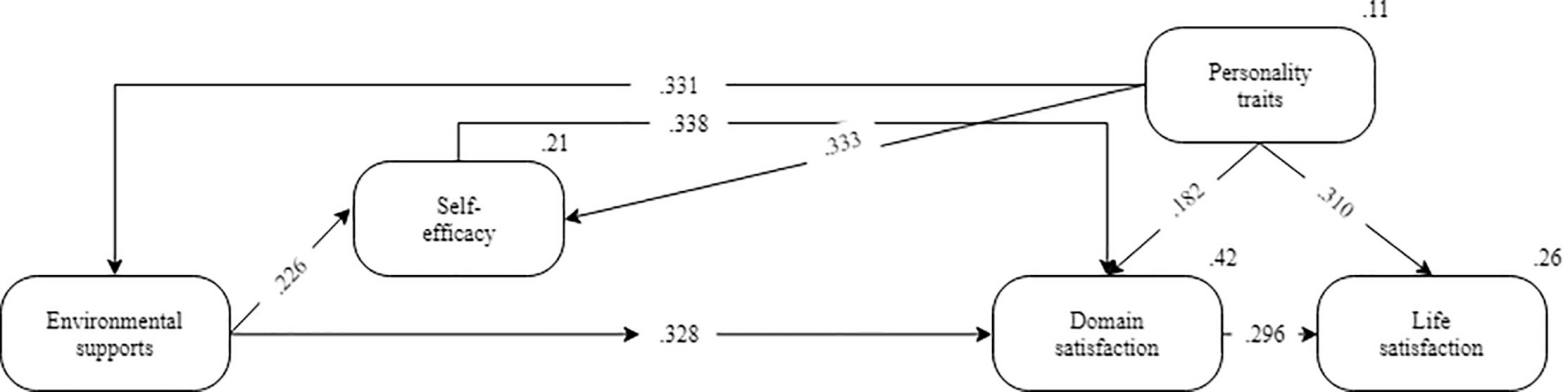
Path analysis for Model II. All paths are significant at *p* < .01.

As revealed in Fig 4, DS is positively associated to life satisfaction (*β* = .296, *p*-value = 0.000). Therefore, hypothesis 1b is supported. This finding is in line with Loewe’s [93] study, which proved DS as an antecedent of life satisfaction. The structural model showed that PTs positively predicted life satisfaction (*β* = .310, *p*-value = 0.000). Thus, hypothesis 1c is supported. This is in line with Ali [94] that PTs may also be key indicators of other facets of individuals’ life, including satisfaction with life. ESs also have a positive correlation with DS (*β* = .328, *p*-value = 0.000). Therefore, hypothesis 2a is supported. Thus, the results are consistent with Lent’s [68] study, which indicated social cognitive variables predicted DS. The data in Fig 4 showed that SE (*β* = .338, *p*-value = 0.000) was positively linked to DS. Hence, hypothesis 2b is supported. It seems that SE could lead to greater DS among the university students. The present study’s findings can confirm Lent’s [68] finding a positive association between SE and DS (*r* = .43). This study revealed that PTs were predictors of DS (*β* = .182, *p*-value = 0.000). Therefore, hypothesis 2e is accepted. This finding supports Watson and Clark’s [95] conclusion that PTs and DS are related. The structural model showed that PTs (*β* = .333, *p*-value = 0.000) were positively associated with SE. Hence, hypothesis 4a is accepted. The findings confirm Navarro’s [96] results, indicating that PTs of engineering students have a direct effect on their SE. As revealed in Fig 4, ESs were significant in justifying the proportion of SE (*β* = .226, *p*-value = 0.000). Thus, hypothesis 4b is accepted. This is in line with Lent and Brown [28], whereby ESs help SE to shape one’s adaptive career behavior (e.g., by helping to regulate skill use and help persistence). The current results showed that PTs could significantly influence ESs (*β* = .331, *p*-value = 0.000). Thus, hypothesis 6a is accepted. This study found similar results with Lent’s [45] study that showed PTs as affective dispositions and dynamic traits, which continually interacted with ESs. Moreover, ESs, SE, and PTs jointly explained 41.9% variance in DS; and PTs and DS explained 26% of the variance in life satisfaction.

## Discussion and conclusions

The study assessed the applicability of SCCT in predicting life satisfaction and also the inter-correlations of SCCT factors by employing a meta-analysis with path analysis. The results did not entirely support the initial SCCT satisfaction model [20]. Thus, GP and OEs are not a factor in the context of higher education students. This confirmed Lent’s [44] study of the non-significant association between OEs and DS. This inconsistency over OEs as explained by Lent [44] is one of the potential issues related to OE measure and/or receiving direct rewards is opposite to the rewards that are expected by students in the long-term, as a greater predictor of DS. The present study excluded OEs (due to model unfit) and this decision is in agreement with Lent [44] and Singley [62] when eliminating OEs. Moreover, the current study also reached the model fit (Model II) by excluding GP. The present results are consistent with Lent’s [68] study, which confirmed the association between GP and DS, but not between GP and ESs. University student samples, which comprised the participants of the present study, might be less likely to testify their engagement in effective pursuit of their goals or unable to be responsive to the types of support (e.g., social, physical, fiscal aspects) that they obtained from the environment, for instance, due to insufficient savings, access to an incomplete variety of career role models, or lack of instrumental support with university applications.

However, with a modified version of the model by removing two constructs, the model was fit for the data. All eight remaining paths created meaningful coefficients, varying in magnitude from small to moderate. The results also yielded theoretically unexpected findings; PTs’ role and their importance in explaining all remaining constructs in the modified SCCT model. The findings proposed that PTs and socio-cognitive factors might not signify distinct, separate causes of satisfaction [97]. Although it is not yet clear that PTs are flexible to modification by psychological intervention, the findings are in line with results from different contexts such as the employees' selection literature that PTs augment overall cognitive ability as main antecedents of life satisfaction [98]. The findings demonstrated the need to incorporate dispositional and socio-cognitive factors and this will be one of the key missions of novel career development theories. Besides, flexible SE proposes a possible objective for intervention attempts since it is realized that a necessary level of ability is also needed to succeed in life satisfaction [99]. SE beliefs to some extent, but not critically, are seen as inspiring students to take on gradually challenging tasks for which success is possible [100]. Findings of the alternative SCCT (Model II) offered support for the view that ESs direct paths to SE as well as DS.

### Limitations and avenues for future studies

The present study provided an organized theory-driven analysis of the studies on SCCT as a path to those embarking on future studies and developing theoretical descriptions. Some limitations must be considered and their possible implications on the findings are deliberated. It is vital to mention that this study like other meta-analyses is limited due to the availability of obtainable findings [101]. When authors do not report adequate statistics in primary studies, meta-analysts cannot include these studies in a MASEM; thus, the information from those primary studies is essentially lost. Meta-analytic results may, though, be anticipated to deliver more strong discoveries than those of single studies. Another limitation of meta-analyses is the bias of publications, i.e., nonpublished studies (e.g., unpublished research studies excluded from the study). Although many factors were incorporated in this study, the number of studies inspected and/or the sample size (n) of these studies was not high because various studies inspected the effect sizes for more than one outcome, and as a result of analyzing the data by using a multi-level modeling approach [102]. Besides, due to the significant heterogeneity of the findings, the accurateness of random effect size was reduced and the small datasets added to this study limited the study’s ability to test moderators that may support the analysis. In the case of insufficient data for conducting a moderator inspection, the particular association was omitted [103]. On the other hand, it is admitted that this was not inevitably the case for concept mapping. However, it is vital to say that in these cases, the studies examined were experimental, gave themselves better recognizing effects, should these exist [104]. Besides, meta-analyses are insensitive to causal directions and not sufficient to infer causality [105]; consequently, longitudinal or experimental and quasi-experimental studies are essential to determine causal associations and make more assured generalizations about the strength of SCCT associations [106]. This is due to the reason that longitudinal studies can capture the relationship among antecedents of LS over time–instead of only as simultaneous predictors of LS at a single time. Along with longitudinal research [107], experimental studies could offer a welcome addition to the literature. Furthermore, intervention research can use the present findings to increase the level of LS among students and other cases. These interventions can be designed to help university students to secure new supports or utilize existing ones that may enable them by strengthening SE, DS, and life satisfaction. For example, it may be likely to help students to assert in part of agency over their affecting regulation. Such a tactic might explain life domains/roles that are of specific relevance to students and evaluate their life satisfaction in these domains/roles. However, these interventions must be offered very cautiously because particular features of well-being (e.g., life satisfaction) may be more changeable and vulnerable to nonpersonality effects than the present study and other studies assumed [108]. Meta-analysis cannot replace focused empirical research in addition to it could not adopt the full complexity of interrelationships between constructs [106, 109]. These interrelationships require to be addressed in prospective studies. They have to study other factors that account for variance in life satisfaction beyond that explained by the SCCT antecedents. The constructs included in this meta-analysis are limited to constructs for which appropriate data are accessible. Therefore, the meta-analysis has to be considered as a summary of the most commonly studied elements of life satisfaction. Future studies may inspect other theories/models and the effects of those variables not comprised in the present study (i.e., positive and negative expected emotions). The present study proposes visions into the pros and cons of theory-driven meta-analysis and meta-analytic structural equation modeling in the area of life satisfaction [110]. A vital area for further meta-analytic research is the potential mediating role of SCCT variables in the relationship between life satisfaction and more distant constructs, like SE and DS. The theory-driven meta-analysis offers a method to address unanswered research questions and reach a sense of theoretical transparency of the relationships that career development researchers strive to understand [111]. Study findings showed that students’ personality traits can predict life satisfaction among university students. Previous studies also showed different results, as some studies found a negative influence of some aspects of personality traits on life satisfaction [112], other studies discovered personality traits as significant predictors of life satisfaction [113]. This inconsistency in previous findings reflects that personality traits are different in every culture and country. Future studies need to consider different cultural contexts when applying the SCCT model. It is also possible for prospective studies to test this model by adding cultural related constructs to see whether the view of LS is different in various countries. This will be helpful to redefine LS and also create novel scales of LS for different cultures.

The population sampled in this meta-analysis was university students. Generalization of the findings to other samples (e.g., high school students, employees, etc.) must be performed cautiously. Regardless of these limitations, this study advises that the modified form of SCCT can offer a usable pattern for comprehending and predicting life satisfaction and designing interventions to satisfy students in their university-to-work transition. Last but not least, it would be useful for prospective studies to test SCCT constructs in predicting life satisfaction of university students, which can be moderated by cultural [114], field-specific, measurement, or sampling considerations.

## Supporting information

S1 Checklist PRISMA 2009 checklist (Adapted for KIN 4400). (DOC)

S1 Fig PRISMA 2009 flow diagram. (TIFF)
