# Supplementary material for: The applicability of social cognitive career theory in predicting life satisfaction of university students: A meta-analytic path analysis
Source: PLoS One. 2020 Aug 21;15(8):e0237838. doi: 10.1371/journal.pone.0237838 (PMC7444545; doi:10.1371/journal.pone.0237838)
Supplement: S1 Checklist — (DOC) [file pone.0237838.s001.doc]

Page 1 of 2

| **Section/topic** | | **#** | | **Checklist item** | **Reported on page #** |
| --- | --- | --- | --- | --- | --- |
| **TITLE** | | | | |  |
| Title | | 1 | | Identify the report as a literature review. |  |
|  | |  | | The applicability of social cognitive career theory in predicting life satisfaction of university students: A meta-analytic path analysis | 1 |
| **ABSTRACT** | | | | |  |
| Structured summary | | 2 | | Provide a structured summary including, as applicable: background; objectives; data sources; study eligibility criteria, participants, and interventions; study appraisal and synthesis methods; results; limitations; conclusions and implications of key findings; |  |
|  | |  | | Background: The university-to-work transition is a vital step in the creation of a job identity for graduates. During this period, some can face this transition confidently, while others experience hesitancy, insecurity, and hopelessness.  Derived from the social cognitive career theory (SCCT), the present study developed a model for the empirical examination of factors affecting life satisfaction of university students.  Methods and Findings: A random-effects meta-analysis of zero-order correlations observed the results of 16 studies (20 samples, n = 7,967), and associations among the SCCT variables were examined by using a meta-analytic structural equation modeling (MASEM) according to a pooled correlation matrix. An alternative model was offered and then assessed. The findings showed a satisfactory fit of the new model as compared to the original SCCT. The results demonstrated support for the alternative model of SCCT in predicting life satisfaction.  Conclusions: The present study suggested that researchers should embrace this alternative model when synthesizing SCCT factors. | Abstract  1, 2 |
| **INTRODUCTION** | | | | |  |
| Rationale | | 3 | | Describe the rationale for the review in the context of what is already known about your topic. |  |
|  | |  | | In this regard, previous research has emphasized that the experience of career development is a critical source for facilitating university students from their uncertain future, resolving unfavorable working conditions and thus increasing their satisfaction with life. Similar research have indicated life satisfaction for university students who are self-assured in their career orientation. From this point of view, it is important to examine how students in the university-to-work transition experience the concomitant successes and failures.  Consequently, there is a lack of theoretical base around university students’ life satisfaction through the university-to-work process and in facilitating their career growth due to the fact that some fundamental theories may not effectively comprise the environmental and contextual elements that contribute to the effects of career development exposure on life satisfaction. | Introduction  2 |
| Objectives | | 4 | | Provide an explicit statement of questions being addressed with reference to participants, interventions, comparisons, outcomes, and study design (PICOS). |  |
|  | |  | | This study aims to test SCCT and how it can predict life satisfaction of university students by using a meta-analytic path analysis. | 3 |
| **METHODS** | | | | |  |
| Eligibility criteria | | 5 | | Specify study characteristics (e.g., PICOS, length of follow-up) and report characteristics (e.g., years considered, language, publication status) used as criteria for eligibility, giving rationale. |  |
|  | |  | | A review protocol was not used for this study. A detailed explanation of the models and datasets including in the study are giving in the methods section and in the Search Strategy section below. | Materials and Methods  10 |
| Information sources | | 6 | | Describe all information sources (e.g., databases with dates of coverage) in the search and date last searched. |  |
|  | |  | | The authors of the present study perused PsycNet, ProQuest, and ScienceDirect to find related research published between January 1, 2004 and December 31, 2018. The combination of words employed to search these records were life, academic, and domain satisfaction, outcome expectations, self-efficacy, environmental and social supports, and social cognitive career theory. Studies met the inclusion criteria if they: (a) employed the SCCT as a theoretical basis, and (b) supplied quantitative data allowing for the calculation of correlation (*r*) and/or effect sizes among the SCCT variables. | 10 |
| Search | | 7 | | Present full electronic search strategy for at least one database, including any limits used, such that it could be repeated. |  |
|  | |  | | As well as exploring journal databases, additional search tactics were used to enlarge the number of studies. The authors investigated the websites of well-known journals with a history of releasing higher education and career studies. They also inspected article references and searched for authors by name. Abstracts were assessed according to the inclusion criteria. | 10 |
| Study selection | | 8 | | State the process for selecting studies (i.e., screening, eligibility). |  |
|  | |  | | First, they excluded duplicated research, studies that involved school students as participants, and qualitative studies. Second, studies were excluded if they did not meet all of the inclusion standards, or if inadequate data were available for calculation. Sixteen studies (20 samples) were chosen for the meta-analytic path analysis (see Fig 2). Finally, Cooper’s method of unstructured search was employed by using Google Scholar and Google for identifying more relevant studies. | 10 |
| Risk of bias in individual studies | | 9 | | Describe methods used for assessing risk of bias of individual studies (including specification of whether this was done at the study or outcome level). | N/A |
| Risk of bias across studies | | 10 | | Specify any assessment of risk of bias that may affect the cumulative evidence (e.g., publication bias, selective reporting within studies). | N/A |
| **RESULTS** | | | | |  |
| Study selection | 11 | | Give numbers of studies screened, assessed for eligibility, and included in the review, with reasons for exclusions at each stage, ideally with a flow diagram. | |  |
|  |  | | The process for study selection is described in the Informational search strategy section above. See Table 1. Strategy for literature search. | | Materials and Methods  10, 11 |
| Study characteristics | 12 | | For each study, present characteristics for which data were extracted (e.g., study size, PICOS, follow-up period) and provide the citations. | |  |
|  |  | | The present study utilized a strategy for data abstraction. The subsequent data were obtained: authors, publication year, aim, country, variables, sample size, and mean age (see Table 3 for further detail). | | 14, 15, and 16 |
| Synthesis of results of individual studies | 13 | | For all outcomes considered (benefits or harms), present, for each study: (a) summary of results and (b) relationship to other studies under review (e.g. agreements or disagreements in methods, sampling, data collection or findings). | |  |
|  |  | | The explicit stated purpose of our analysis was to assess SCCT and its applicability among higher education students. Therefore, we did not assess bias in individual studies. Instead, we assessed heterogeneity. The *I^2^* statistic was employed to determine the degree of heterogeneity. The estimated heterogeneity variances explained in Table 5. | | 17, 20 |
| **DISCUSSION** | | | | |  |
| Summary of evidence | 14 | | Summarize the main findings including the strength of evidence for each main outcome; consider their relevance to key groups (e.g., healthcare providers, users, and policy makers). | |  |
|  |  | | The study assessed the applicability of SCCT in predicting life satisfaction and also the inter-correlations of SCCT factors by employing a meta-analysis with path analysis. The results did not entirely support the initial SCCT satisfaction model.  However, with a modified version of the model by removing two constructs, the model was fit for the data. All eight remaining paths created meaningful coefficients, varying in magnitude from small to moderate. | | Discussion  Paragraph 1, 2  P.24 |
| Limitations | 15 | | Discuss limitations at study and outcome level (e.g., risk of bias), and at review-level (e.g., incomplete retrieval of identified research, reporting bias). | |  |
|  |  | | Limitations of our study explained explicitly in Limitation and avenues for future studies. There are three type of limitations which were methodological, practical, search strategy limitations. We included some tips and methods for future studies to handle these challenges. | | 25, 26, 27 |
| **CONCLUSION** | | | | |  |
| Conclusions | 16 | | Provide a general interpretation of the results in the context of other evidence, and implications for future research. | |  |
|  |  | | The study assessed the applicability of SCCT in predicting life satisfaction and also the inter-correlations of SCCT factors by employing a meta-analysis with path analysis. The results did not entirely support the initial SCCT satisfaction model.  However, with a modified version of the model by removing two constructs, the model was fit for the data. All eight remaining paths created meaningful coefficients, varying in magnitude from small to moderate. The results also yielded theoretically unexpected findings; personality traits role and their importance in explaining all remaining constructs in the modified SCCT model. The findings proposed that personality traits and socio-cognitive factors might not signify totally distinct, separate causes of satisfaction. Although it is not yet clear that personality traits are flexible to modification by psychological intervention, the findings are in line with results from different contexts such as the employees selection literature that personality traits augment overall cognitive ability as main antecedents of life satisfaction. The findings demonstrated the need to incorporate dispositional and socio-cognitive factors and this will be one of the key missions of novel career development theories. In addition, self-efficacy, which is flexible, proposes a possible objective for intervention attempts since it is realized that a necessary level of ability is also needed to succeed in life satisfaction. Self-efficacy beliefs to some extent, but not critically, are seen as inspiring students to take on gradually challenging tasks for which success is possible. Findings of the alternative SCCT (Model II) offered support for the view that environmental supports direct paths to self-efficacy as well as domain satisfaction. | | 24, 25 |

*Adapted from:*  Moher D, Liberati A, Tetzlaff J, Altman DG, The PRISMA Group (2009). Preferred Reporting Items for Systematic Reviews and Meta-Analyses: The PRISMA statement*. PLoS Medicine*, 6(6), e1000097. doi:10.1371/journal.pmed1000097

For more information, visit: **www.prisma-statement.org**.

Page 2 of 2
